# Optimization of FRET imaging in *Arabidopsis* protoplasts

**DOI:** 10.1016/j.mocell.2025.100180

**Published:** 2025-01-10

**Authors:** Gyuho Choi, Yerim Cha, Tae-Jin Kim, Gah-Hyun Lim

**Affiliations:** 1 Department of Integrated Biological Science, College of Natural Sciences, Pusan National University, Busan 46241, Republic of Korea; 2 Department of Biological Sciences, College of Natural Sciences, Pusan National University, Busan 46241, Republic of Korea; 3 Institute of Systems Biology, Pusan National University, Busan 46241, Republic of Korea

**Keywords:** Calcium biosensor D3cpv, Förster resonance energy transfer, Förster resonance energy transfer biosensor, Image post processing, Protoplast fluorescence imaging

## Abstract

Recent advancements in fluorescence-based biosensor technologies have enabled more precise and accurate Förster resonance energy transfer (FRET) imaging within *Agrobacterium*-mediated plant transformation systems. However, the application of FRET imaging in plant tissues remains hindered by significant challenges, particularly the time-intensive process of generating transgenic lines and the complications arising from tissue autofluorescence. In contrast, protoplast-based FRET imaging offers a rapid and efficient platform for functional screening and analysis, making it an essential tool for plant research. Nevertheless, conventional protoplast-based FRET approaches are often limited by background interference, inconsistent imaging conditions, and difficulties in quantitative analysis. Here, we present a systematic optimization of imaging conditions using the calcium biosensor D3cpv, addressing these limitations to improve both precision and efficiency in protoplast-based FRET imaging. This work serves as a practical guide for streamlining FRET imaging workflows and maximizing the utility of biosensors in plant cell studies.

## INTRODUCTION

Förster resonance energy transfer (FRET) is a widely utilized and powerful technique for studying molecular interactions and dynamic processes in living cells with high spatiotemporal resolutions (Haustein et al., 2003, Hochreiter et al., 2015, Verma et al., 2023). While FRET has been extensively applied in animal systems, its adoption in plant biology has encountered significant challenges. Autofluorescence in plant cells, mainly from chlorophylls and secondary metabolites, creates significant challenges for FRET analyses by generating background signals that interfere with accurate fluorescence measurements. Chlorophyll is the main source, emitting strongly at 670 nm and overlapping with the spectra of many fluorescent proteins, especially blue-light-excited ones like cyan fluorescent protein (Müller et al., 2013, Roshchina, 2003, Roshchina, 2012). This spectral interference remains a critical barrier, as it reduces the accuracy of signal detection and hampers the reliability of FRET measurements (Donaldson, 2020, García-Plazaola et al., 2015, Kodama, 2016). Despite these limitations, advancements in biosensors and fluorescent proteins specifically optimized for plant systems are helping to mitigate these issues. These innovations, combined with improvements in imaging techniques, are gradually overcoming autofluorescence-related challenges and enhancing the precision of FRET applications in plants (Banerjee et al., 2016, Bücherl et al., 2014, Denay et al., 2019, DeVree et al., 2021, Dixit et al., 2006, Hamers et al., 2014, Long et al., 2018, Terryn et al., 2018). This study aims to optimize FRET-based sensors for use in protoplast systems, providing a robust platform for precise and reliable monitoring of cellular dynamics in plant cells. Specifically, by utilizing the calcium biosensor D3cpv, the efficiency and accuracy of FRET imaging in *Arabidopsis* protoplasts have been significantly enhanced. This optimized approach serves as a valuable tool in plant biology for investigating key molecular processes, including stress responses and signaling pathways.

## THE PRINCIPLE OF FRET IMAGING

FRET imaging is based on the nonradiative energy transfer between 2 fluorophores: a donor and an acceptor molecule, when they are in close proximity (typically within 10 nm) (Lakowicz, 2006). FRET occurs when the emission spectrum of the donor overlaps with the excitation spectrum of the acceptor, allowing energy transfer through dipole-dipole interactions. The detection of FRET signals relies on the measurement of changes in fluorescence intensity or lifetime. In donor-acceptor pairs, FRET results in the quenching of donor fluorescence and the simultaneous enhancement of acceptor fluorescence. These changes can be quantified using fluorescence microscopy techniques, such as fluorescence intensity-based microscopy, fluorescence lifetime imaging microscopy, or radiometric analysis (Algar et al., 2019, Periasamy et al., 2015).

## PROTOPLAST ISOLATION AND D3CPV CALCIUM BIOSENSOR TRANSFECTION

**Fig. 1:**
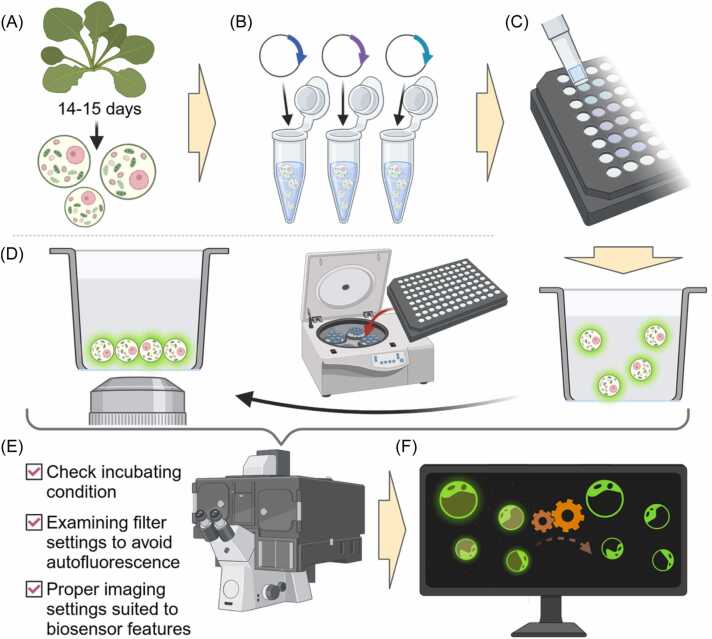
Overview of the protoplast imaging. (A-D) Protoplasts were isolated from the leaves of 14 to 15-day-old *Arabidopsis thaliana* plants and transfected with the appropriate amount of plasmid DNA. The transfected protoplasts were subsequently positioned at the bottom of 96-well plate using a centrifuge. (E) Imaging conditions and setting tailored to protoplast. (F) Postprocessing software ensures noise reduction and reliable data acquisition. Schematic drawing produced by BioRender (http://biorender.com/).

*Arabidopsis* plants were grown in an environment-controlled chamber with a photoperiod of 14 h of light and 10 h of dark at a constant temperature of 21°C, under light conditions (150-190 μE m^−2^ s^−1^) and 55% to 60% relative humidity (Yoo et al., 2007). The leaves from 14 to 15-day-old plants are treated with Cellulase R-10 and Macerozyme R-10 enzyme solution to digest the cell wall, followed by filtration and centrifugation to isolate the protoplasts (Zhai et al., 2009). The isolated protoplasts are then suspended in MMG solution (0.4 M mannitol, 15 mM MgCl_2_, 4 mM MES) and subjected to transfection using the respective plasmids (Fig. 1A). To enhance transfection efficiency, a cell density of 10^6^ cell/ml and 30 µg of plasmid DNA encoding the D3cpv were used (Fig. 1B).

## PREPARATION OF PROTOPLAST SAMPLES FOR IMAGING IN MICROPLATES

Glass-bottom microplates provide superior optical clarity and reduce background interference, thereby enhancing the quality of fluorescence imaging. To ensure reliable results during protoplast preparation, precise volume control minimizes disturbances within the wells. A high protoplast density during suspension dilution can lead to aggregation in the 96-well plate, causing overlapping fluorescence signals and degraded image quality. To achieve an optimal monolayer formation and prevent aggregation, approximately 50,000 protoplasts per well are recommended (Fig. 1C). The protoplasts can be gently sedimented to the bottom of the microplate by centrifugation at 500 rcf for 10 min using a microplate carrier (Fig. 1D). Once positioned, protoplasts require a stable ambient temperature, as they are highly sensitive to fluctuations. Consistent laboratory conditions or the use of incubators help maintain this stability (Fig. 1E).

## PREPARING PROTOPLAST IMAGE ACQUISITION AND POST PROCESSING

**Fig. 2:**
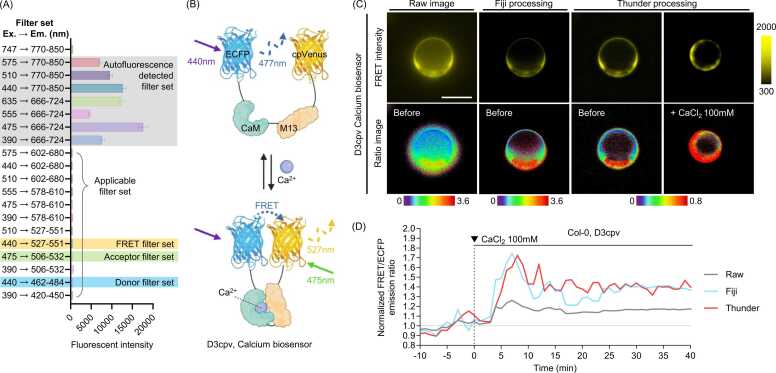
Enhancing quantitative analysis for protoplast imaging through autofluorescence avoidance and post processing. (A) Autofluorescence in protoplasts was analyzed across filter sets using a Leica DMi8 microscope with an LED8 light source. Excitation and emission wavelengths define each filter set. Gray shading indicates autofluorescence, while yellow, green, and cyan shading mark the FRET, acceptor, and donor channels, respectively. (B) Schematic representation of the calcium biosensor D3cpv. Upon calcium binding, calmodulin interacts with the M13 peptide, causing a conformational change that brings the fluorescent proteins ECFP and circularly permuted Venus closer together. Excitation at 440 nm leads to energy transfer (FRET) from ECFP to circularly permuted Venus, resulting in emission at 527 nm. In the absence of calcium, FRET efficiency decreases, and ECFP emission at 477 nm is dominant. (C) Image postprocessing reduces background noise and clarifies ratio images. FRET ratio images were created by dividing FRET by donor images in Fiji and processed further using Thunder in Las X software (Leica). Protoplasts from wild-type Col-0 were transfected with the D3cpV calcium biosensor. Postprocessed images show enhanced FRET ratio clarity, with warmer colors indicating higher FRET/ECFP ratios. Scale bar = 20 µm. (D) Postprocessing increases sensitivity in FRET data. The graph depicts normalized FRET/ECFP ratio changes over time induced by CaCl_2_, processed using Fiji and Thunder from the same raw data. ECFP, enhanced cyan fluorescent protein.

Autofluorescence significantly complicates the accurate measurement of fluorescence by distorting signals. To confirm the presence of autofluorescence, nontransfected protoplasts were used to evaluate the autofluorescence emitted across all fluorescent filter combinations. Subsequently, it was assessed whether the filter set responsible for autofluorescence overlapped with the filter set intended for the fluorescent protein in the biosensor to be used. If an overlap was detected, alternative strategies, such as substituting the fluorescent protein or employing a different filter set, were considered (Figs. 1E and 2A). D3cpv is a genetically encoded calcium indicator based on FRET between 2 fluorophores, enhanced cyan fluorescent protein as the donor and circularly permuted Venus as the acceptor. This biosensor contains a calmodulin domain and a M13 peptide, which undergo conformational changes upon calcium binding, leading to changes in FRET efficiency. As calcium levels increase, the interaction between calmodulin and M13 brings enhanced cyan fluorescent protein and circularly permuted Venus into closer proximity, enhancing energy transfer and enabling real-time calcium quantification in protoplasts (Fig. 2B). Each biosensor exhibits varying levels of reactivity depending on its specific application, requiring optimization of filter settings, exposure time, intervals, and imaging duration during initial experiments (Fig. 1E). Even after protoplasts are spin down to the bottom of the imaging chamber, their spherical shape can produce fluorescent noise signals originating from out-of-focus regions. These background signals may hinder the precise differentiation of objects when generating ratio images. However, this issue can be partially mitigated through postimage processing (Fig. 1F).

For image post processing, various plugins available in the Fiji software package (Fiji/ImageJ), such as DeconvolutionLab2 (EPFL Biomedical Imaging Group) and MOSAICsuite (MOSAIC group), were utilized. These tools are freely accessible and have been widely endorsed by prior studies (Sage et al., 2017, Schindelin et al., 2012, Shivanandan et al., 2013). Specifically, FRET and donor images were deconvolved using the DeconvolutionLab2 plugin with the Richardson-Lucy algorithm for 10 iterations. The point-spread function was generated using the Richards and Wolf point-spread function model as the optical model, tailored to the specific wavelength of each channel. After deconvolution, background signals were removed using the "Plugin > Mosaic > Utility > Background Subtractor" feature. In addition, commercial software such as Leica's Lightning or Thunder modes was employed to further reduce background signals effectively. Thunder processing was performed using the large volume computational clearing mode. Reducing background signals significantly enhances the clarity of ratio and intensity images, enabling precise data collection from specific objects and resulting in more reliable outcomes (Fig. 2C and 2D).

## CONCLUDING REMARKS

This study highlights the potential of optimized FRET imaging in Arabidopsis protoplasts as a robust platform for plant cellular analysis. By addressing challenges such as autofluorescence and background interference, we significantly enhanced imaging precision and sensitivity using the D3cpv calcium biosensor. Postprocessing techniques, including Fiji and Thunder software, further improved data quality and reliability. These advancements offer a practical guide for efficient biosensor application, providing valuable insights into molecular dynamics in plant systems. Future efforts should focus on adapting these techniques for in vivo studies to expand their applicability in plant biology.

## FUNDING AND SUPPORT

This work was supported by the National Research Foundation of Korea (NRF-2022R1C1C1012729) and (NRF-2022R1A4A5031503) and 2023 BK21 FOUR Program of Pusan National University.

## AUTHOR CONTRIBUTIONS

**Gyuho Choi**, **Yerim Cha**, **Tae-Jin Kim**, **Gah-Hyun Lim:** Conceptualization. **Gyuho Choi**, **Yerim Cha:** Writing – original draft preparation. **Tae-Jin Kim**, **Gah-Hyun Lim:** Supervision, Project administration, Funding acquisition.

## DECLARATION OF COMPETING INTERESTS

The authors declare that they have no known competing financial interests or personal relationships that could have appeared to influence the work reported in this paper.

## References

[bib1] Algar W.R., Hildebrandt N., Vogel S.S., Medintz I.L. (2019). FRET as a biomolecular research tool-understanding its potential while avoiding pitfalls. Nat. Methods.

[bib2] Banerjee S., Garcia L.R., Versaw W.K. (2016). Quantitative imaging of FRET-based biosensors for cell-and organelle-specific analyses in plants. Microsc. Microanal..

[bib3] Bücherl C.A., Bader A., Westphal A.H., Laptenok S.P., Borst J.W. (2014). FRET-FLIM applications in plant systems. Protoplasma.

[bib4] Denay G., Schultz P., Hänsch S., Weidtkamp-Peters S., Simon R. (2019). Over the rainbow: a practical guide for fluorescent protein selection in plant FRET experiments. Plant Direct.

[bib5] DeVree B.T., Steiner L.M., Głazowska S., Ruhnow F., Herburger K., Persson S., Mravec J. (2021). Current and future advances in fluorescence-based visualization of plant cell wall components and cell wall biosynthetic machineries. Biotechnol. Biofuels..

[bib6] Dixit R., Cyr R., Gilroy S. (2006). Using intrinsically fluorescent proteins for plant cell imaging. Plant J..

[bib7] Donaldson L. (2020). Autofluorescence in plants. Molecules.

[bib8] García-Plazaola J.I., Fernández-Marín B., Duke S.O., Hernández A., López-Arbeloa F., Becerril J.M. (2015). Autofluorescence: biological functions and technical applications. Plant Sci..

[bib9] Hamers D., van Voorst Vader L., Borst J.W., Goedhart J. (2014). Development of FRET biosensors for mammalian and plant systems. Protoplasma.

[bib10] Haustein E., Jahnz M., Schwille P. (2003). Triple FRET: a tool for studying long-range molecular interactions. ChemPhysChem.

[bib11] Hochreiter B., Pardo Garcia A., Schmid J.A. (2015). Fluorescent proteins as genetically encoded FRET biosensors in life sciences. Sensors.

[bib12] Kodama Y. (2016). Time gating of chloroplast autofluorescence allows clearer fluorescence imaging in planta. PLoS One.

[bib13] Lakowicz J.R. (2006). Principles of fluorescence spectroscopy.

[bib14] Long Y., Stahl Y., Weidtkamp-Peters S., Smet W., Du Y., Gadella T.W., Goedhart J., Scheres B., Blilou I. (2018). Optimizing FRET-FLIM labeling conditions to detect nuclear protein interactions at native expression levels in living Arabidopsis roots. Front. Plant Sci..

[bib15] Müller S.M., Galliardt H., Schneider J., Barisas B.G., Seidel T. (2013). Quantification of Förster resonance energy transfer by monitoring sensitized emission in living plant cells. Front. Plant Sci..

[bib16] Periasamy A., Mazumder N., Sun Y., Christopher K.G., Day R.N. (2015). FRET microscopy: basics, issues and advantages of FLIM-FRET imaging. Adv. Time-Correl. Single Photon Count. Appl..

[bib17] Roshchina V.V. (2003). Autofluorescence of plant secreting cells as a biosensor and bioindicator reaction. J. Fluoresc..

[bib18] Roshchina V.V. (2012). Vital autofluorescence: application to the study of plant living cells. Int. J. Spectrosc..

[bib19] Sage D., Donati L., Soulez F., Fortun D., Schmit G., Seitz A., Guiet R., Vonesch C., Unser M. (2017). DeconvolutionLab2: An open-source software for deconvolution microscopy. Methods.

[bib20] Schindelin J., Arganda-Carreras I., Frise E., Kaynig V., Longair M., Pietzsch T., Preibisch S., Rueden C., Saalfeld S., Schmid B. (2012). Fiji: an open-source platform for biological-image analysis. Nat. Methods.

[bib21] Shivanandan A., Radenovic A., Sbalzarini I.F. (2013). MosaicIA: an ImageJ/Fiji plugin for spatial pattern and interaction analysis. BMC Bioinformatics.

[bib22] Terryn C., Paës G., Spriet C. (2018). FRET-SLiM on native autofluorescence: a fast and reliable method to study interactions between fluorescent probes and lignin in plant cell wall. Plant Methods.

[bib23] Verma A.K., Noumani A., Yadav A.K., Solanki P.R. (2023). FRET based biosensor: principle applications recent advances and challenges. Diagnostics.

[bib24] Yoo S.D., Cho Y.H., Sheen J. (2007). Arabidopsis mesophyll protoplasts: a versatile cell system for transient gene expression analysis. Nat. Protoc..

[bib25] Zhai Z., Sooksa-nguan T., Vatamaniuk O.K. (2009). Establishing RNA interference as a reverse-genetic approach for gene functional analysis in protoplasts. Plant Physiol..

